# Comprehensive Psychopathological Assessment Based on the Association for Methodology and Documentation in Psychiatry (AMDP) System: Development, Methodological Foundation, Application in Clinical Routine, and Research

**DOI:** 10.3389/fpsyt.2017.00045

**Published:** 2017-04-07

**Authors:** Rolf-Dieter Stieglitz, Achim Haug, Erdmann Fähndrich, Michael Rösler, Wolfgang Trabert

**Affiliations:** ^1^Department of Psychology, Clinical Psychology and Psychiatry, Psychiatric University Hospital, University Basel, Basel, Switzerland; ^2^Clienia-Group, University of Zürich, Zürich, Switzerland; ^3^Psychiatric Hospital of the Vivantes Klinikum Neukölln, Berlin, Germany; ^4^University of the Saarland Neurocentre, Homburg, Saarland, Germany; ^5^Department of Psychiatry, Psychotherapy and Psychosomatics, Klinikum Emden, Emden, Germany

**Keywords:** Association for Methodology and Documentation in Psychiatry system, psychopathology, observer rating scale, assessment, AMDP-System, documentation

## Abstract

The documentation of psychopathology is core to the clinical practice of the psychiatrist and clinical psychologist. However, both in initial as well as further training and specialization in their fields, this particular aspect of their work receives scanty attention only. Yet, for the past 50 years, the Association for Methodology and Documentation in Psychiatry (AMDP) System has been in existence and available as a tool to serve precisely the purpose of offering a systematic introduction to the terminology and documentation of psychopathology. The motivation for its development was based on the need for an assessment procedure for the reliable documentation of the effectiveness of newly developed psychopharmacological substances. Subsequently, the AMDP-System began to be applied in the context of investigations into a number of methodological issues in psychiatry (e.g., the frequency and specificity of particular symptoms, the comparison of rating scales). The System then became increasingly important also in clinical practice and, today, represents the most used instrument for the documentation of psychopathology in the German-speaking countries of Europe. This paper intends to offer an overview of the AMDP-System, its origins, design, and functionality. After an initial account of the history and development of the AMDP-System, the discussion will in turn focus on the System’s underlying methodological principles, the transfer of clinical skills and competencies in its practical application, and its use in research and clinical practice. Finally, potential future areas of development in relation to the AMDP-System are explored.

## Introduction

Psychopathology has enjoyed a long tradition in psychiatric literature [e.g., Kraepelin, Bleuler, Jaspers, Schneider; cf. Ref. (1, 2)]. Semple et al. (3), p. 80, describe this area of specialization in the following terms: “Psychopathology is the study of abnormalities in mental states and is one of the core sciences in clinical psychiatry. Descriptive psychopathology is one method for describing the subjective experience and behavior of patients and is the basis for our current clinical descriptions of mental disorder.” In recent years, interest in the systematic discussion of psychopathological questions has waned. One possible reason for this may be the introduction of operationalized diagnosis by classification systems such as the World Health Organization’s ICD-10 (since 1992) and the American Psychiatric Association’s DSM-III (1980; current version: DSM-5 since 2013). An aspect which remains persistently ignored in the context of these classification systems is the fact that most of their diagnostic criteria consist of psychopathological symptoms, and a firm command of a comprehensive body of psychopathological knowledge and expertise is, therefore, an essential requirement for establishing a clinical diagnosis based on either of these systems.

Descriptions of psychopathological phenomena of patients’ experiences and behaviors are found in earliest editions of systematic textbooks of psychiatry and psychology. The philosopher Karl Jaspers, in his general psychopathology (German title: *Allgemeine Psychopathologie*), laid the foundation for psychopathology as a scientific discipline in its own right. Since then, psychopathology has featured as the essential basic core subject in the study of psychiatry and clinical psychology. The discovery and development of psychotropic drugs during the mid-1950s prompted a widening of the discussion on psychopathological issues. For the first time in the history of psychiatry, new forms of therapy were able to effect rapid changes in a patient’s mental state. This triggered the need for precise and accurate recording of such medication-induced changes in a patient in order to accurately and comprehensively track and document a patient’s development throughout a given course of treatment. Psychotropic drugs opened up entirely new opportunities for differential therapies in the treatment of psychopathological disorders. As a result, differential diagnosis became an absolute prerequisite for effective differential therapy in the field of psychiatry also, as was already the case in all other medical disciplines. The first versions of a number of rating scales were designed [cf. Association for Methodology and Documentation in Psychiatry (AMDP) and CIPS, 1990], such as the Present State Examination (PSE) Scale, the Brief Psychiatric Rating Scale (BPRS), the Inpatient Multidimensional Psychiatric Scale (IMPS), and the Comprehensive Psychiatric Rating Scale (CPRS). These developments coincided with the creation also of the AMDP-System. While most first-generation assessment scales and procedures (e.g., PSE, IMPS, CPRS) have since been superseded and are now rarely applied or persist in modified form only (BPRS), the AMDP-System continues to enjoy widespread acceptance and use in its largely unaltered original form, predominantly in the German-speaking countries of Europe.

## History of the AMDP-System

In 1952, the two French psychiatrists, Delay and Deniker, published their clinical findings on the calming, stabilizing effects of chlorpromazine [cf. Ref. (4)] and thus initiated the development of modern psychotropic substances. This, in turn, laid the foundation for a new scientific discipline: the discipline of psychopharmacology, which led, *inter alia*, to Kuhn and Janssen’s respective discoveries of the antidepressant effect of imipramine and the antipsychotic drug haloperidol [cf. Ref. (5)]. During that time, numerous new substances were undergoing clinical trials. While the effectiveness of these new substances was clearly observable, the objective assessment of such findings was rendered difficult in the absence of the scientific instruments necessary for this purpose.

In order to counter this problem, two working groups formed independently of each other at university clinics in Germany and Switzerland. Their common objective was to design and develop an instrument that would enable the systematic recording and assessment of the effects of new psychotropic drugs on individual patients. Subsequent close cooperation between the two groups ultimately led to the formation of the AMDP [cf. Ref. (6)]. The purpose of the intended instrument was not only to facilitate the documentation of medical histories of psychiatric patients but also to offer a comprehensive overview of psychopathological and somatic symptoms with the specific aim of facilitating the exchange of information in relation to psychiatric medical histories, assessments, and findings at an international level. The resulting instrument has since undergone further improvement and development to accommodate findings from widening clinical experience as well as from an increasingly large body of empirical studies (e.g., a reduction of the number of items listed in the overview of psychopathological symptoms from 123 to 100 and of somatic symptoms from 58 to 40). Since 1979, the instrument has been commonly known and referred to as the AMDP-System (7). In Germany, besides finding application as a research instrument, the AMDP-System soon also began to serve as the basis for routine medical reports on admission and discharge at several psychiatric university clinics. Training seminars are primarily used to improve the quality of the collected data (interrater reliability) in research as well as in clinical routine.

In addition to engaging in the creation and development of the AMDP-System, the AMDP working group began to organize symposia for the purpose of addressing methodological issues in psychiatry (e.g., questions concerning the recording and assessment of changes observed in patients under treatment), developing supplementary modules (e.g., in relation to negative symptoms), and promoting the translation of the AMDP-System into different languages. In 1976, Daniel Bobon (Liège) established an international secretariat (8). The AMDP Manual was translated from its original German version into various other languages, including Danish, Japanese, Croatian, Dutch, Russian, Greek, and Italian (8). In 1980, an English-speaking AMDP working group was established, and the publication of a first English edition of the AMDP-System followed 2 years later (9). A new, updated English version of the AMDP Manual is due for publication in 2017^1^; also published resp. planned are editions in Portuguese, French, and Norwegian. Another clear indication of the AMDP working group’s commitment to an international focus is also the publication of a further manual in collaboration with the CIPS (Collegium Internationale Psychiatriae Scalarum) on the application and use of rating scales in psychiatry. This manual contains references to the 16 self-rating and observer-rating scales most commonly applied in psychiatric studies and research (along with the AMDP-System, these include e.g., the Hamilton Scale (HAMD), the BPRS, the Bech-Rafaelsen Mania Scale, and the BRMS; also the Clinical Global Impression rating scale, CGI). Each of these scales is reproduced in the manual in four languages (English, French, Italian, and German).

Since 1988, the AMDP working group has been further committed to the training of psychiatrists and psychologists in descriptive psychopathology (e.g., by regularly organizing multi-day training seminars in this field).

The year 2016 saw the publication of the ninth revised German edition of the AMDP-System with an additional section on optionally applicable new symptoms. For psychopathological findings, these are *panic attack, acceleration of thinking, ideas of reference, disorganized behavior, lack of boundaries, thought hearing, impulsive behavior, disturbance of body image, feelings of shame, overvalued ideas*, and *anomic aphasia*; for somatic findings, they are *parasomnia, increased libido*, and *sexual disturbances*. This ninth revised edition of the AMDP-System will also form the basis for its new, forthcoming foreign-language editions.

## Methodological Aspects of the AMDP-System

Since its inception, the AMDP working group has been, as its name suggests, extensively concerned with methodological issues relating to the documentation and diagnosis in psychiatry. Its initial goal, however, was the development of a rating scale with particular focus on the following three aspects:

1. precise definitions of psychopathological terms/symptoms;
2. clear definitions of assessment criteria for individual psychopathological phenomena;
3. and the operationalization of assessment procedures for individual psychopathological phenomena.

These objectives were born out of the observation that psychopathological terms often tended to be interpreted and applied in an inconsistent, imprecise manner. The AMDP Manual, the result of a development process covering several years, offers clear, precise *definitions* of 100 psychopathological and 40 somatic symptoms and of a number of supplementary, optionally applicable symptoms for both of these categories (11 symptoms and 3 symptoms, respectively). Individual entries for each of the symptoms listed in the manual adhere to the same structure, as follows:

- definition
- explanations and examples
- notes on rating
- symptoms to differentiate from.

Table 1 below contains an extract from the AMDP Manual in the form of such an entry. A short, concise definition of the symptom is followed by more in-depth and descriptive details in order to lead to a better understanding of the term defined (e.g., likely examples of a patient’s verbal utterances). Psychopathological phenomena, as far as their presence or absence in a patient is concerned, manifest by degree rather than on an all-or-nothing basis. Assessment advice given for a particular symptom focuses on descriptions of the degrees of severity of “mild” and “severe.” Both these levels are intended as threshold levels at which the recording of manifestations of a symptom as “mild” or “severe” becomes justified. The section on “Symptoms to differentiate from” is intended to offer further assistance in determining the potential presence or absence of a particular symptom.

**Table 1:** **Structure of an Association for Methodology and Documentation in Psychiatry (AMDP) symptom description: example**.

**34. Delusional perception (S)**	
*Definition*	
Abnormal significance/meaning, usually related to one’s self, is attributed to real perceptions. This happens without the presence of a rationally or emotionally understandable underlying reason. This symptom represents a delusional misinterpretation of an accurate perception of an external stimulus.	
*Explanations and examples*	
During a walk in the park, a patient notices a dog looking at him and lifting one of its front paws. The patient interprets this as a sign of divine revelation.	
“Mnestic delusional perceptions,” as particular forms of *delusional perception*, are also documented here: “When I was a kid I had a fork which had this symbol of a crown engraved on it. Now I suddenly realise what that was meant to mean – I’m clearly of noble descent!”	
Instances of mistaken identity are also recorded here as long as the act of recognition is of genuine character (i.e., meets the criteria for a *delusional perception*).	
Instances of *delusional perception* reported and recorded prior to the current period of assessment are classified as *36. Delusional ideas* and documented there	
*Notes on rating*	
“Mild”	Instances of *delusional perception* are limited to specific areas of experience (one instance of reported *delusional perception* within the current period of assessment).
“Severe”	The patient’s entire experience is dominated by *delusional perceptions* (in excess of three different instances of *delusional perception* reported within the current period of assessment).
*Symptoms to differentiate from*	
35. Sudden delusional ideas	
36. Delusional ideas	
47. Illusions	

A clear definition of a specific symptom, however, does not suffice as long as the *basis on which to make an assessment* remains unclear. The following are considered potential sources of data on which to base an assessment: the patient, the rater (i.e., the examining physician or clinician), and third parties (e.g., care staff, family members, and close relatives). These potential sources each assume varying degrees of significance in connection with the assessment of individual psychopathological symptoms. Some psychopathological symptoms can be reliably assessed exclusively on the basis of a patient’s personal account (e.g., in the case of hallucinations), while other symptoms are observable only by the rater or third parties with the patient remaining consciously unaware of being afflicted with any form of disorder (e.g., a formal disorder of thought such incoherence or neologisms). With a third group of symptoms, assessment is possible by the patient as well as third parties (e.g., lack of drive). In view of these differences, each symptom has been allocated one of the following data source categories:

- S (=self) The patient’s own personal account or description of a particular experience, occurrence, or set of circumstances is required.
- O (=other) The examiner’s observation of a particular occurrence or set of circumstances is required or deemed appropriate and sufficient.
- SO (=self *or* other)

Both types of data source should be taken into consideration for assessment, whereby a positive assessment of a particular experience, occurrence, or set of circumstances by one data source alone is deemed sufficient to positively confirm the presence of the symptom in question.

**Figure 1:**
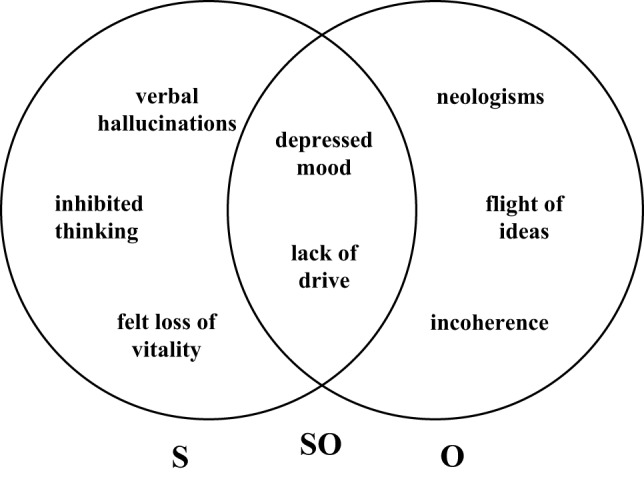
**Data sources for Association for Methodology and Documentation in Psychiatry (AMDP) symptoms (S, self; O, other; SO, self or other)**.

Figure 1 attempts to illustrate some examples.

**Figure 2:**
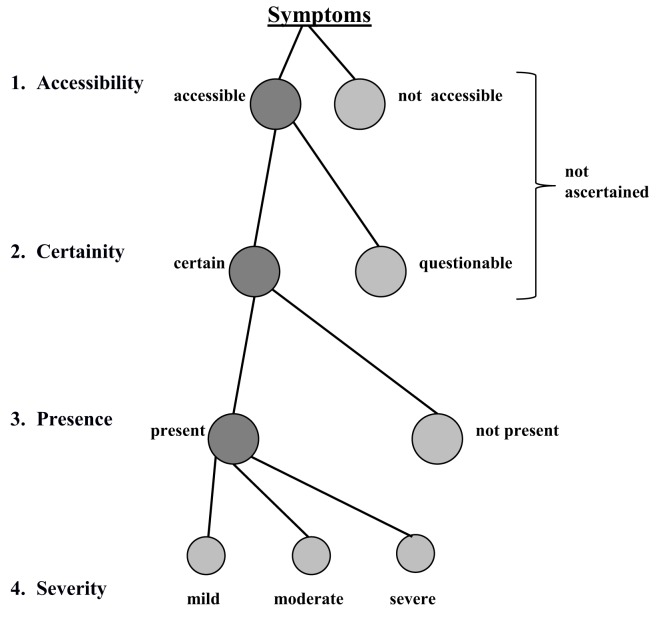
**The Association for Methodology and Documentation in Psychiatry (AMDP) decision tree**.

One of the most notable innovations introduced by the AMDP working group, however, is the so-called *AMDP decision tree*, which details the sequence of steps necessary in the decision process for the assessment of each symptom (Figure 2).

The first level of the AMDP decision tree (accessibility) requires a decision to be made as to whether or not it is possible for a particular symptom to be reliably assessed. Symptoms for disorders of perception, for example, cannot be assessed where a patient refuses to communicate with the examiner or refuses to communicate in general or fails to respond adequately to the examiner’s prompts and questions. Such behavior, however, tends to be rare. More commonly observed are instances where a patient supplies insufficient information for reliable assessment of a particular symptom (the second level of the AMDP decision tree). At the third level of the AMDP decision tree, in cases where sufficient and reliable information is forthcoming and available, it remains for the examiner to make the final decision as to the presence or absence of a particular symptom. For a symptom to be assessed as not present, the requirement for a sufficient amount of information on which to base such assessment must be equally fully met. Where a symptom has been assessed as present, the degree of severity with which the symptom presents must then be rated (the fourth level of the AMDP decision tree). Only this graduation makes it possible to record the treatment process in a differentiated way (treatment evaluation).

**Figure 3:**
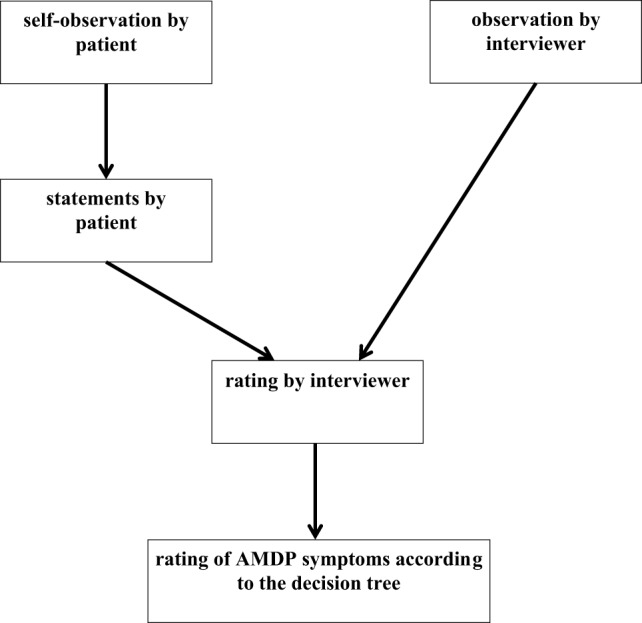
**Pathway to decision within the Association for Methodology and Documentation in Psychiatry (AMDP) System**.

It is important to remember that the AMDP-System is, in essence, *an observer rating scale* (as, e.g., the HAMD rating scale). Although the patient’s own statements are clearly indispensable and an explicit requirement for the assessment of most symptoms, the ultimate decision as to whether to assess a specific symptom as present or not *always* rests exclusively with the examiner alone. Figure 3 illustrates this point clearly.

**Figure 4:**
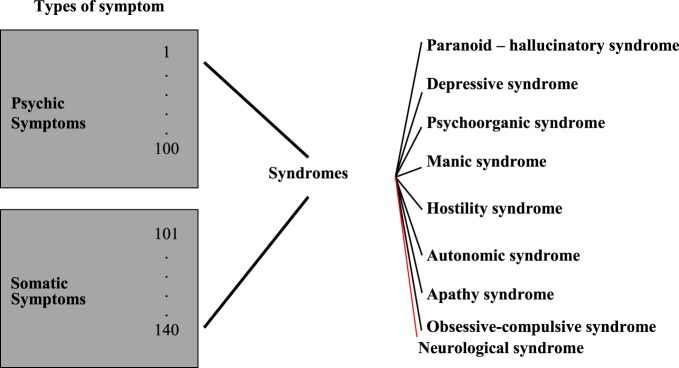
**Development of the Association for Methodology and Documentation in Psychiatry (AMDP) syndromes**.

A further important aspect of the AMDP-System was the development of syndrome scales. Not long after the publication of the AMDP Manual, first studies were conducted into devising syndrome or rating scales based on the AMDP-System pool of symptoms (10). Syndrome scales (observer rating scales) do not provide any diagnoses. They allow the quantification of the severity of a certain syndrome (e.g., depressive syndrome). The currently valid syndromes based on the AMDP-System are represented in Figure 4 and Table 2 below (11).

**Table 2:** **Association for Methodology and Documentation in Psychiatry (AMDP) syndromes (11)**.

AMDP syndromes	Example: manic syndrome (MANI)
Paranoid-hallucinatory syndrome Depressive syndrome Psychoorganic syndrome Manic syndrome Hostility syndrome Autonomic syndrome Apathy syndrome Obsessive–compulsive syndrome Neurological syndrome	22. Flight of ideas 66. Euphoria 72. Exaggerated self-esteem 82. Increased drive 83. Motor restlessness 88. Logorrhea 93. Excessive social contact

These AMDP Syndrome Scales contain a further spectrum of clinically relevant syndromes (e.g., depressive syndrome, paranoid-hallucinatory syndrome) and have been validated in a large number of studies and applied in numerous research projects, mostly in the field of psychopharmacology (12).

## Clinical Training

Training in psychopathology, despite being of primary importance, remains often patchy, unsystematic, and unstructured (13). Although psychopathology regularly features as a prescribed, compulsory staple in all psychiatric–psychotherapeutic curricula, compared with the quality of training offered in other psychotherapeutic areas of therapy, it remains a subject treated as of secondary importance only and pushed to the margins. While both ICD and DSM have now operationalized psychiatric disorders by means of strict and clearly defined diagnostic criteria, they fail to define the psychopathological terminology on which these operationalization criteria are based. Clearly, there is a general unstated assumption that the user of these classification tools is thoroughly familiar with psychopathological terminology and fully capable of correctly defining, for example, the term *delusional perception* – a symptom which, to this day, remains core to a diagnosis for schizophrenia (Table 1).

The AMDP-System with its lists of 100 psychopathological, 40 somatic, and 14 optionally applicable symptoms and their respective definitions offers an extremely broad (albeit not entirely concise) range of options to describe abnormal behavior and experience in a patient. The advantages to be gained from such a stock of symptoms and their definitions are manifold.

– Specific terms cannot be randomly defined and applied, since their definition is clearly stated in the manual. Each application of a specific term must be plausibly justifiable with reference to the definition provided for it.
– All 140 symptoms must be assessed. This ensures the recording also of symptoms which may not be spontaneously reported by the patient, and the decision as to which symptoms to enquire about and which to leave out during examination is not left to the sole discretion of the individual examiner. This further enables also clear statements to be made about symptoms assessed as not present. The recording on the AMDP-System documentation form of a particular symptom as “not ascertained” automatically indicates a mandatory requirement for further examination at a later stage, thus preempting the risk of individual symptoms being inadvertently overlooked during initial examination.
– An interview guide (14) is available for working with the AMDP-System. However, instead of a standardized format, the assessment procedure follows a semi-structured approach in which all symptoms must be explored as to whether they were experienced (S) or observed (O), to arrive at a judgment. The order in which the questions are asked may be freely chosen and their contents adapted to the requirements of the interview situation.

The following concrete example illustrates the potential for information gaps in psychopathological descriptions in cases where the focus remains directed at primary symptoms only (an extract from findings on admission for a female patient):

Awake, fully conscious. Fully orientated. Reduced attention span and poor memory. Retarded thinking. Suffers from the delusion of being persecuted. Experiences verbal hallucinations. Suspected visceral or visual hallucinations, although patient denies experiencing these. The patient experiences emotional despair and markedly reduced drive. The patient reports sporadic experience of suicidal thoughts from which she is, however, able to credibly distance herself during examination.

On recording these details on the AMDP-System documentation form for psychopathological findings (which lists 100 symptoms), statements corresponding to only 19 of the symptoms listed in the AMDP-System are found. As regards the remaining 81 of these symptoms, the fact whether the lack of mention of them in this set of findings is due to the symptoms having been assessed as not present, or due to not having been assessed at all, remains entirely open to speculation. Unfortunately, the latter generally tends to be the case.

In recent years, the AMDP-System has been increasingly deployed as an instrument in the training of practitioners in assessing and recording psychopathological findings. Almost all German text books on the subject are based on the symptoms and the definitions contained in the Manual, an invaluable compendium which has also found its way into texts and commentaries on projects relating to national planning policy on public health and well-being and health infrastructure.

Training in the assessment and documentation of psychopathological findings lends itself particularly well to group practice sessions. Over 30 years’ experience of regularly organized AMDP training seminars has been accumulated to date (13). Current seminars normally take place in groups of around 20 participants and are led by 2 AMDP-System-trained and -experienced presenters. By means of live or video-recorded examination interviews conducted by the presenters with three different patients, three case studies with three different symptomatologies are introduced which then each form the subject of subsequent in-depth discussions. After each examination interview, seminar participants are challenged to rate all identified symptoms based on the corresponding AMDP-System documentation form. This is then followed by a guided debate on what at this stage usually amounts to symptom ratings of considerable variance among individual participants. Each participant is required to substantiate the rating of a particular symptom with examples of actual observed patient utterances or behavior, or with other observations made during interview. Participants are asked to adhere rigorously to the definitions contained in the AMDP Manual, to differentiate strictly between factual symptom observation and symptom interpretation and to resist the temptation to engage in any form of diagnostic assessment at this stage. It has been possible to show that within the course of just a single seminar, interrater-reliability is noticeably improved and participants’ knowledge base of psychopathological terminology demonstrably enhanced (15–17). It is imperative for such group coaching in psychopathology to occur at regular intervals. As a rule, such sessions are easily integrated in in-house training programs at individual clinics (case conferences). Only in this way can the continued validity and effectiveness of the AMDP-System terminology be safeguarded and maintained.

The AMDP-System recording form for psychopathological findings, on which individual symptoms are rated, must be regarded as an essential, yet not wholly self-sufficient assessment tool. The AMDP-System operates on the premise that its purpose is not to act as a substitute for the freely formulated assessment report on psychopathological findings. Ultimately, it is this report which conveys an authentic picture of the assessed patient as a person. The value of the completed AMDP-System documentation form, however, lies in serving as an excellent basis from which to draw for the formulation of clinical findings and for the structuring of case discussions.

Intermittent critics have voiced their concern that the application of a standardized system of the type advocated here curtails too strictly the flexibility and adaptability necessary to accurately document human experience and behavior. Such concerns are justified, albeit in the case of the AMDP-System somewhat misguided. The AMDP-System is far from claiming to be a comprehensive authority in the recording of all human experience and behavior. Yet, it offers a stock of clear and accessible terminology to draw from, in order to facilitate the compilation of such records and reports. By way of analogy, the AMDP-System may be likened to an essentially lifeless string instrument, which must first be mechanically tuned and intonation techniques be mastered before it allows for an inexhaustible range of original musical tunes to be successfully elicited from it. In other words, where psychopathological terms and definitions are unclearly formulated and inappropriately used (analogically speaking, a badly tuned string instrument played with poor intonation), the contents of a report on psychopathological findings (analogous to the musical tunes) cannot be rendered lively and precise in the best of circumstances.

Another point of criticism occasionally leveled against the AMDP-System is the length of time required to assess and rate the 100 psychopathological symptoms listed. With sufficient practice, this process should, as a general rule, take up no more than 45 min. It must be remembered, however, that such assessment procedure is never limited to the recording of psychopathological findings alone but always simultaneously marks either the beginning or an intensification of a therapeutic relationship between examiner and patient. Patients are appreciative of sincere enquiries about their needs and expectations as well as their medical history and personal well-being. Such enquiries must be conducted in a sensitive and respectful manner, and in essence, they amount to what patients ultimately expect from their psychologists and psychiatrists by way of standard good practice.

Diametrically opposed to the above point of criticism is the objection to the fact that the AMDP-System contains a mere 100 symptoms, which some rate as wholly inadequate to properly reflect the varied and multifaceted nature of human experience. This objection is entirely warranted, and it is by no means the intention of the AMDP-System to limit examiners to these 100 symptoms, prohibiting them from going beyond that figure and documenting further symptoms in addition to them. Reality, however, appears to indicate a tendency among examiners toward the opposite, as was exemplified by the extract from a set of psychopathological findings on admission reproduced earlier in this text. Practical experience shows that the number of symptoms being examined for tends more likely to be insufficient rather than adequate or even excessive.

A final and, to some extent, anachronistic charge levied against the AMDP-System arises from questioning the need for the differentiated documentation of psychopathological findings [for general discussion, see Ref. (18–22)]. Ultimately, so the argument goes, most clinical pictures are, as characteristically reoccurring patterns of particular disorders, easily recognizable at practically first sight. It was exactly this blind belief in the reliability of “pattern recognition,” however, which led to the introduction of operationalized diagnostic criteria, since the perceived validity of that method was ultimately revealed as a myth. Attempts at developing neurobiological methods as reliable diagnostic markers have so far proved equally unsuccessful. Quite the contrary: clearly, neurobiological research is heavily reliant on the most precise phenotype descriptions possible in order to be able to create a homogeneous sample in the first place. Diagnostic assessments and decisions on therapies have always been based on psychopathological findings and will continue to be so for the foreseeable future. Against this background, unclear terminology can never result in a clear diagnosis. A case in point is “delusional perception.” The term “delusional perception” (Table 1) was described by Kurt Schneider (with reference to Jaspers and Gruhle) as a primary symptom for the diagnosis of schizophrenia. According to ICD-10 coding algorithms, this symptom continues to this day to be of enormous importance in diagnosing schizophrenia. What exactly, however, is “delusional perception”? ICD-10 contains no descriptive details for the term. Textbooks frequently explain instances of “delusional perception” in terms of true perceptions to which false, delusional meanings are attributed. What remains also often ignored or forgotten is Kurt Schneider’s caveat that for “delusional perception” to be diagnosed accurately, such attachment of delusional meaning to an otherwise accurate perception must occur without rationally or emotionally justifiable underlying cause. Delusional or delusion-like misinterpretations of true perceptions are also frequently present in the absence of a schizophrenic disorder, e.g., during episodes of severe depression accompanied by psychotic symptoms in the context of experiencing intense feelings of guilt; with social phobia against the background of intense feelings of shame; and with paranoid personality disorder as a result of extreme distrust (suspiciousness). In all these cases, an emotional trigger for misinterpretation is indicated, and yet, none is representative of an authentic case of “delusional perception.” Only with an exact awareness of how “delusional perception” is defined, and after precise and thorough analysis as to whether an observed phenomenon accurately corresponds with this definition or not, does a valid diagnosis become possible.

Descriptive psychopathology is a sub-category of general psychopathology. Psychopathology as a science is not merely concerned with the exact description of unusual experiences and behavior but addresses a considerably wider range of aspects (hermeneutic, biographical, etc.). Without precise descriptive psychopathology, however, all psychiatric findings (and the conclusions and decisions based on them) remain arbitrary and ambiguous. Against this background, the AMDP-System with its terminology based on classic psychopathology makes an important contribution to education and training in the field of psychopathology. By continuously reiterating to the user the need to differentiate between carefully observed and described phenomena on the one hand and their interpretation as symptoms on the other, it forestalls hasty decision-making and circular reasoning. Only in this way can an initial diagnosis be reviewed and, where appropriate, alternative hypotheses developed. Diagnostic sub-groups can be identified which, in turn, opens up opportunities to investigate these homogeneous sub-groups with other (e.g., neurobiological) methods in the context of further research. A disregard for precise psychopathological description, however, leads any neurobiological research to failure, since such research will lack connection to clinical reality and become self-referential. First warning bells to this effect began to be sounded as far back as the 1990s [“Cassandra’s complaints” (21)].

“Fortunately, the Europeans still have a proud tradition of clinical research and descriptive psychopathology. Someday, in the 21^st^ century, after the human genome and the human brain have been mapped, someone may need to organize a reverse Marshall plan so that the Europeans can save American science by helping us figure out who really has schizophrenia or what schizophrenia really is (23), p. 1407.

We need to make a serious investment in training a new generation of real experts in the science and art of psychopathology. Otherwise, we high-tech scientists may wake up in 10 years and discover that we face a silent spring. Applying technology without the companionship of wise clinicians with specific expertise in psychopathology will be a lonely, sterile, and perhaps fruitless enterprise [(24), p. 1639].”

As is common knowledge, Troy fell and Cassandra’s prophetic cries went unheeded. Yet, just as Virgil once, in his Aeneid, picked up the thread of Homer’s Iliad anew, so there is today a fresh, powerful drive underway which seeks to defend and afford psychopathology anew its ever relevant and irrefutably proper place in the fields of psychiatry and clinical psychology as the essentially fundamental and indispensable science that it indisputably represents (22, 25).

## The AMDP-System and Its Application in Research and Clinical Routine

### Evaluation of the AMDP-System As an Assessment Tool

The publication of the AMDP-System enabled fundamental methodological critique in psychiatry to be performed for the first time. It became possible to examine the psychopathological terms defined in the AMDP-System based on psychometric criteria. In essence, the primary purpose of these investigations was to determine whether the fairly complex psychopathological phenomena emanating from traditional psychiatry allowed for assessment in such a way that different raters arrived at concurring results regarding the psychopathological state of a given patient. Were they to fail in this, the attempt to devise assessment scales for symptoms of a high degree of complexity would have to be abandoned. A further objective of the evaluation process was to examine the relation of the AMDP-System to other assessment procedures (especially other observer rating scales such as the IMPS and CPRS). In the field of psychology, with the application of psychological testing procedures, strategies of this kind had long become the established norm. In psychopathology, by contrast, investigating for objectivity, reliability, and validity was uncharted territory.

In the context of reliability studies, the question of *interrater-reliability* was of primary concern (26, 27). During the years when the AMDP-System was being developed and published, ensuring reliability and comparability of psychopathological findings and diagnoses was core. With regard to the *validity* of AMDP-System-based findings, especially questions on convergent and divergent validity were examined. An example of this was the comparison of the AMDP-System Syndrome Scale for “depressive syndrome” with the Bech-Rafaelsen Melancholia Scale and the Beck Depression Inventory (28). Only moderate correlation was found between observer-rating scales and self-rating scales. Results of investigations into the psychometric properties of the instrument, including factor analyses and the classification of findings in the tradition of psychiatry, are summarized in Berner (29), Pietzcker et al. (11), Baumann et al. (10), and Heimann and Rein (26).

### The AMDP-System As an Assessment Tool for Methodological Questions

After extensive and thorough evaluation, the AMDP-System began to be applied in the examination of various methodological issues. Thus, the AMDP-System has always also served as a tool for external validation of other instruments (9). Previous teams of authors compared various approaches to the development of assessment scales from different countries. Short specialist scales [e.g., the HAMD, the BPRS, and the Clinical Global Impressions (CGI), etc.] were discussed in comparison with the AMDP-System, which is representative for a comprehensive, multifaceted global scale. In this context, in their exemplary study, Theodoridou et al. (30) investigated the concurrent validity and sensitivity to change of the 12-item Health of Nation Outcome Scales and the 3-item CGI in relation to the 100-item AMDP-System, which the authors used as a psychopathological reference scale. The correlations between the instruments were significant and plausible.

Further investigations centered around the frequency and specificity of psychopathological symptoms, the grouping of symptoms into syndromes (see above), and the classification of the latter into specialist nosological concepts (11, 29). A comparison of data from various sources led to a careful differentiation between self-rating and observer-rating procedures in the psychopathological examination process.

More recent studies focused on the empirical structure of psychopathological findings and comparisons between dimensional and categorical diagnostic concepts in the application of AMDP data (31–33). In many instances, these studies relied on the method of multidimensional scaling for their enquiries and were able to fall back on earlier factor-analytic approaches. Cuesta and Peralta (34) investigated 660 patients suffering from an acute psychotic episode and found a 10-dimensional model by principal component analysis including 67 items of the AMDP-System. The comparison of this factor solution with current psychopathological AMDP data (31), carried out on a large random sample with multidimensional scaling in two-dimensional symptom cards, yielded clusters which were largely identical with the symptom areas resulting from earlier factor-analytic evaluations. Although some of the symptom clusters described at the time amounted to projections only which were neither formally confirmed nor established, the consistency of psychopathological symptom structures found over a period of some 20 years remained impressive.

### The AMDP-System As an Evaluation Tool for Clinical Proof of Concept

Given the underlying objectives guiding the development of the AMDP-System as an instrument for the documentation and assessment of the effectiveness of psychopharmacological intervention, clinical-pharmacological studies play an important role (12, 15). Studies on the effectiveness of antidepressants and antipsychotics are now widely available (35, 36), and the effectiveness of benzodiazepines was investigated by Ansseau et al. (37).

Reischies et al. (38) discussed the combined treatment of acute mania. The relevance of the AMDP-System “Apathy Syndrome” scale in the measuring of the effectiveness of intervention was investigated by Reischies and Stieglitz (39). Occasionally, somatic findings contained in the AMDP-System are applied separately in order to document the side effects of pharmacotherapy [e.g., Ref. (40, 41)].

Volz et al. (42) chose as their subject of investigation the effects of various forms of light therapy on the symptoms of depressed patients.

### The AMDP-System in Clinical Studies

On account of its broad range of descriptions of psychopathological phenomena, the AMDP-System is encountered in many clinical studies in the context of a range of distinctly diverse issues, as the following paragraphs illustrate (Table 3). Given the relatively concentrated focus on schizophrenic and affective disorders during the development of the AMDP-System, most available studies center around aspects of these two disorders. Based on the example of schizophrenic disorders, Cuesta and Peralta (34) demonstrated the need to combine neurobiological studies with differentiated psychopathological assessment instruments such as the AMDP-System.

**Table 3:** **Selection of pharmacological interventions and clinical studies referring to schizophrenia, bipolar, and depressive disorders by use of the Association for Methodology and Documentation in Psychiatry (AMDP) System**.

Study type	Reference
**Psychopharmacological interventions**	
Validity of the AMDP-System for its use in clinical psychopharmacology	Angst and Woggon (12)
Effect of neuroleptics on positive and negative symptoms and the deficit state	Angst et al. (35)
Pilot study of PK 11195, a selective ligand for the peripheral-type benzodiazepine-binding sites in patients with anxious or depressive symptomatology	Ansseau et al. (37)
The tolerability and efficacy of the atypical neuroleptic remoxipride compared with clozapine and haloperidol in acute schizophrenia	Klieser et al. (36)
Initial triple therapy of acute mania, adding lithium and valproate to neuroleptics	Reischies et al. (38)
**Clinical studies**	
Schizophrenia	
Prevalence of alcohol and drug abuse in schizophrenic inpatients	Soyka et al. (43)
Cannabis and schizophrenia: results of a follow-up study	Caspari et al. (44)
Insight dimensions and cognitive function in psychosis: a longitudinal study	Cuesta et al. (45)
The differentiation between “lack of insight” and “dysfunctional health beliefs” in schizophrenia	Linden and Godemann (46)
Social disability in schizophrenic, schizoaffective, and affective disorders	Bottlender et al. (47)
Long-term term outcome of schizoaffective disorder. Are there any differences with respect to schizophrenia?	Pinna et al. (48)
Bipolar disorder	
Hallucinations in bipolar disorder: characteristics and comparison to unipolar depression and schizophrenia	Baethge et al. (49)
Prevalence of delusional jealousy in psychiatric disorders	Soyka and Schmidt (50)
Depression	
The importance of psychosocial factors, gender, and severity of depression in distinguishing between adjustment and depressive disorders	Barnow et al. (51)
Typus melancholicus personality structure and the characteristics of major depressive episode	Stanghellini et al. (52)
AMDP profiles predict later risk for criminal behavior and violent crimes in former inpatients with affective disorder	Soyka and Zingg (53)
The validity of self-rated psychotic symptoms in depressed inpatients	Seemüller et al. (54)
Depression with psychotic features is influenced by polymorphism of the serotonin transporter gene	Stamm et al. (55)

Caspari (44) investigated the relationship between *schizophrenia* and cannabis consumption, while Soyka et al. (43) looked into alcohol and drug abuse among patients with schizophrenia. Frequency levels of hallucinations co-occurring with schizophrenia, bipolar disorder, and unipolar depression formed the subject of enquiry in Baethge et al. (49). In a comparative longitudinal study of schizophrenic and schizoaffective patients, Pinna et al. (48) addressed issues surrounding the documentation of symptom regression and functional remission in both types of disorder. The effects of schizophrenic, schizoaffective, and affective disorders on social functionality were investigated by Bottlender et al. (47). Möller et al. (56) used the AMDP-System as an outcome criterion in their longitudinal study into schizophrenic and affective disorders. A specialist psychopathological enquiry focused on the question of levels of lack of insight with schizophrenic patients (57). In their longitudinal study, Cuesta et al. (45) were able to demonstrate that a patient’s cognitive performance had insignificant bearing on insight dimensions. Linden and Godemann (46) in their investigation into lack of insight (also assessable with the AMDP-System) and delusional convictions in response to enquiries addressing personal health and well-being with schizophrenic patients were able to present these issues as two distinct constructs. The relationship between delinquency and violence in schizophrenic patients formed the subject of a study which Soyka et al. (58) undertook by applying the AMDP-System. They found that high score values in relation to hostility syndrome and manic syndrome in schizophrenic and bipolar patients may be indicative of an increased risk of criminal behavior (50, 53). Delusions of jealousy is a relatively rare symptom in patients suffering from schizophrenia and other psychotic disorders. Such patients are considerably more likely to display a tendency toward aggression and violence (50).

The significance of psychotic symptoms in *depressed patients* formed the subject of two further studies (54, 55), while Barnow et al. (51) investigated the degree of impact exercised by levels of severity of *depressive* disorders. Zahn et al. (59) enquired into the significance of self-blame and feelings of worthlessness in the context of depressive disorders. Stanghellini et al. (52) applied the AMDP-System in their study into typus melancholicus.

The AMDP-System has also been used in connection with other psychiatric disorders. Bidzan et al. (60), for example, investigated the psychopathological syndrome of *Alzheimer’s* disease. Krasnianski et al. (61) applied the AMDP-System in their description of prevalence rates and natural progression of psychopathological symptoms over time in the case of *Creutzfeld-Jakob* disease. In a longitudinal study on patients suffering from *Parkinson’s* disease who had undergone subthalamic nucleus stimulation, Drapier et al. (62) used the AMDP-System to evaluate symptoms of anxiety. The psychopathological patterns found in eating disorders were looked at by Speranza et al. (63).

In the context of currently evolving demographic changes in population structure, the work by Braca et al. (64) on the psychopathology of *migrants* is of particular interest. In their estimation, the AMDP-System with its wide spectrum of psychopathology is of specific relevance in this context, as evidenced by Diefenbacher and Heim (65) study on Turkish patients (1994).

### The AMDP-System in Everyday Clinical Practice

In Germany, the AMDP-System forms the basis for the documentation of psychopathological findings and quality control in a number of psychiatric clinics (66) and is generally applied in the context of examinations on admission and discharge. Comparability of psychopathological findings is being ensured through regular attendance of training seminars by those in charge of documenting such findings. The AMDP-System is also increasingly regularly used as a module in the documentation of findings in electronically stored patient medical histories. Equally, the AMDP-System is gaining ground in the area of psychiatric expert testimony.

## Conclusion and Perspectives

A profound grasp of descriptive psychopathology and high levels of proficiency in its practical application form part of the most fundamental skills set of a psychiatrist or psychologist. Compared to training courses in psychotherapy, the minimalist approach adopted toward descriptive psychopathology in the design and delivery of training courses in psychiatry is surprising, to say the least, given that descriptive psychopathology represents the bedrock of operationalized diagnosis for psychiatric disorders and the planning of targeted therapy. While in the field of psychotherapy the general expectation of the experienced psychiatrist or psychologist is that they would have spent a considerable number of years studying psychotherapeutic procedure, initial psychiatric-psychological training includes a cursory introduction to descriptive psychopathology only.

Since the first beginnings of psychopharmacology over 50 years ago, however, the AMDP-System has been available as a comprehensive and continuously improved and updated observer-rating scale, enabling the description of a multitude of different facets of psychopathology. The AMDP-System has been translated into several major languages, and since its first publication in 1971 it has, alongside its clinical application, been used in a growing number of studies. Not only does the AMDP-System facilitate the differentiated documentation of findings at symptom level but also enables a differentiated description of the most important psychiatric syndromes in the form of AMDP Syndrome Scales. The latest edition of the AMDP-System distinguishes itself from earlier ones by offering further precision in its descriptions as well as an extension of the range of symptoms described, thus opening up opportunities for still wider clinical application and new areas of research.

The development of the new edition of the DSM originally triggered an extremely critical international discussion about the notion of illness in psychiatry and the meaningfulness of a widely extended range of diagnoses. From this has grown widespread skepticism toward psychiatric classification *per se*. In a climate of such skepticism, an instrument such as the AMDP-System, which, rather than being based on conceptual diagnostic categories, is oriented toward the fundamental phenomenology of experience and behavior, carries within it great potential for improvements in clinical practice as well as for innovation in research.

Especially in times of increasing awareness of the biological basis underlying psychiatric disorders and considering also the steadily increasing stream of new developments in imaging techniques and genetic procedures, there is now more than ever a call for a system like the AMDP-System. Against the background of such new levels of awareness and technical advances in diagnostic procedures, the AMDP-System, with its express focus on the smallest unit of psychiatric observation, i.e., the symptom, represents the key to a much needed and increasingly essential correlation of technical findings with experience and behavior of the patients themselves. Against this background, the AMDP-System coincides entirely with tendencies in current research such as the Research Domain Criteria (67), which define the differentiated and precise documentation of the smallest possible single phenomena as the initial target and the basis upon which to conduct subsequent further analyses of a more complex nature. The AMDP-System undoubtedly answers the requirements in support of this approach in full and thus re-affirms and cements its position as the cornerstone of effective and comprehensive psychopathological assessment as well as of training in psychiatry and clinical psychology at all levels.

## Author Contributions

R-DS, EF, MR, and WT drafted parts of the manuscript and critically revised the entire manuscript. AH critically revised the entire manuscript.

## Conflict of Interest Statement

The authors declare that the research was conducted in the absence of any commercial or financial relationships that could be construed as a potential conflict of interest.

## Footnotes

^1^Broome M, Bottlender R, Rösler M, Stieglitz R-D. *The AMDP-System*. Göttingen: Hogrefe (in press).
